# Ovarian carcinosarcoma with lung metastasis characterized by persistent fever: A case report and literature review

**DOI:** 10.1097/MD.0000000000040202

**Published:** 2024-10-25

**Authors:** Weijing Wang, Xuechao Ji, Hanchao Yang, Xinbo Wang

**Affiliations:** a Department of Obstetrics and Gynecology, Affiliated Hospital of Shandong Second Medical University, School of Clinical Medicine, Shandong Second Medical University, Weifang, China; b Department of Obstetrics and Gynecology, Affiliated Hospital of Shandong Second Medical University, Weifang, Shandong, China; c Department of Gynecologic Oncology, Beijing Obstetrics and Gynecology Hospital, Capital Medical University, Beijing Maternal and Child Health Care Hospital, Beijing, China; d Department of Pathology, Affiliated Hospital of Shandong Second Medical University, Weifang, Shandong, China; e Department of Obstetrics and Gynecology, Shandong Provincial Maternal and Child Health Care Hospital Affiliated to Qingdao University, Jinan, China.

**Keywords:** fever of unknown origin, lung metastasis, malignant mixed Müllerian tumor, neoplastic fever, ovarian carcinosarcoma

## Abstract

**Rationale:**

Ovarian carcinosarcoma (OCS) is a rare malignant tumor prone to distant metastasis. Primary manifestations include pelvic and/or abdominal pain, bloating, and compression. Nevertheless, it is uncommon for OCS to present primarily with persistent fever. This is the first reported case of OCS with lung metastasis characterized by persistent fever.

**Patient concerns:**

A 61-year-old female patient complaining of abdominal pain and fever was admitted to our hospital. Computed tomography showed an irregular, slightly low-density mass on the left side of the uterus and multiple solid nodules in both lungs.

**Diagnoses:**

She underwent cytoreductive surgery for pathologically confirmed stage IVB OCS.

**Interventions:**

She was administered chemotherapy after cytoreductive surgery. Given the patient’s history of persistent fever and progressively enlarged pulmonary nodules, a pulmonary abscess was considered as a possible diagnosis. Following antibiotic therapy, the patient’s high body temperature did not decrease; however, following nonsteroidal anti-inflammatory drug therapy, it quickly decreased. These symptoms were eventually considered the consequence of neoplastic fever caused by lung metastases.

**Outcomes:**

Owing to the rapid progression of the disease, the patient ultimately died.

**Lessons:**

This study suggests that, for patients with pelvic and/or abdominal pain, bloating, and pelvic masses, especially those with suspicious lesions in other organs accompanied by fever of unknown origin, a diagnosis of cancer or sarcoma with metastasis should be considered after ruling out infectious fever.

## 1. Introduction

Ovarian carcinosarcoma (OCS), also known as malignant mixed Müllerian tumor, comprises both cancerous (malignant epithelial) and sarcomatous (mesenchymal) components. According to the dualistic theory of ovarian carcinogenesis, ovarian cancer is a type II carcinoma, and <5% of ovarian cancer cases are caused by this uncommon, invasive epithelial cancer. OCS often occurs at an advanced stage and most frequently metastasizes to the liver, followed by the lungs, bones, and brain.^[1]^ Common presenting features of OCS include pelvic and/or abdominal pain, bloating, and compression symptoms. Nevertheless, fever as the primary or sometimes the initial symptom is uncommon in women with OCS.

Fever of unknown origin (FUO) can be classified into the following 4 categories based on its etiology: infections, malignancies, autoimmune conditions, and miscellaneous. Neoplastic fever (NF) is a paraneoplastic syndrome that arises from the cancer itself and is frequently responsible for FUO in individuals with cancer. NF is related to the release of pyrogenic cytokines. Frequent symptoms of NF include irregular fever, feeling of warmth, perspiration without chills, ineffective antibiotic therapy, and positive response to nonsteroidal anti-inflammatory drugs. There have been reports of several cancers, including lymphoma, lung cancer, colon cancer, and pancreatic carcinoma, first presenting as NF^[2–5]^; however, the relationship between OCS and NF has not been previously reported.

NF is a diagnosis of exclusion that is usually determined after a detailed evaluation of the patient and the exclusion of other etiologies of fever. Consequently, fever in patients with cancer typically presents as a diagnostic conundrum. In this case report, we report on a 61-year-old female patient who was diagnosed with OCS and lung metastasis, which was mainly characterized by persistent fever.

## 2. Case presentation

**Figure 1.**
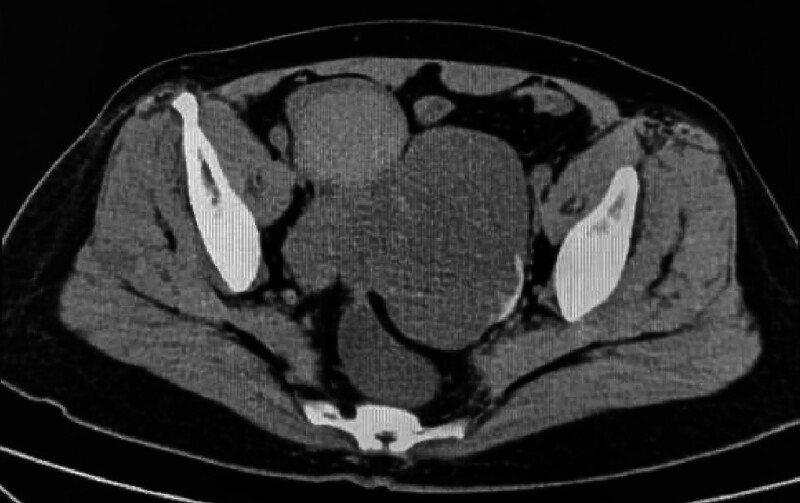
Abdominal computed tomography shows an irregular slightly low-density mass measuring 9.8 × 7.9 × 7.2 cm on the left side of the uterus.

**Figure 2.**
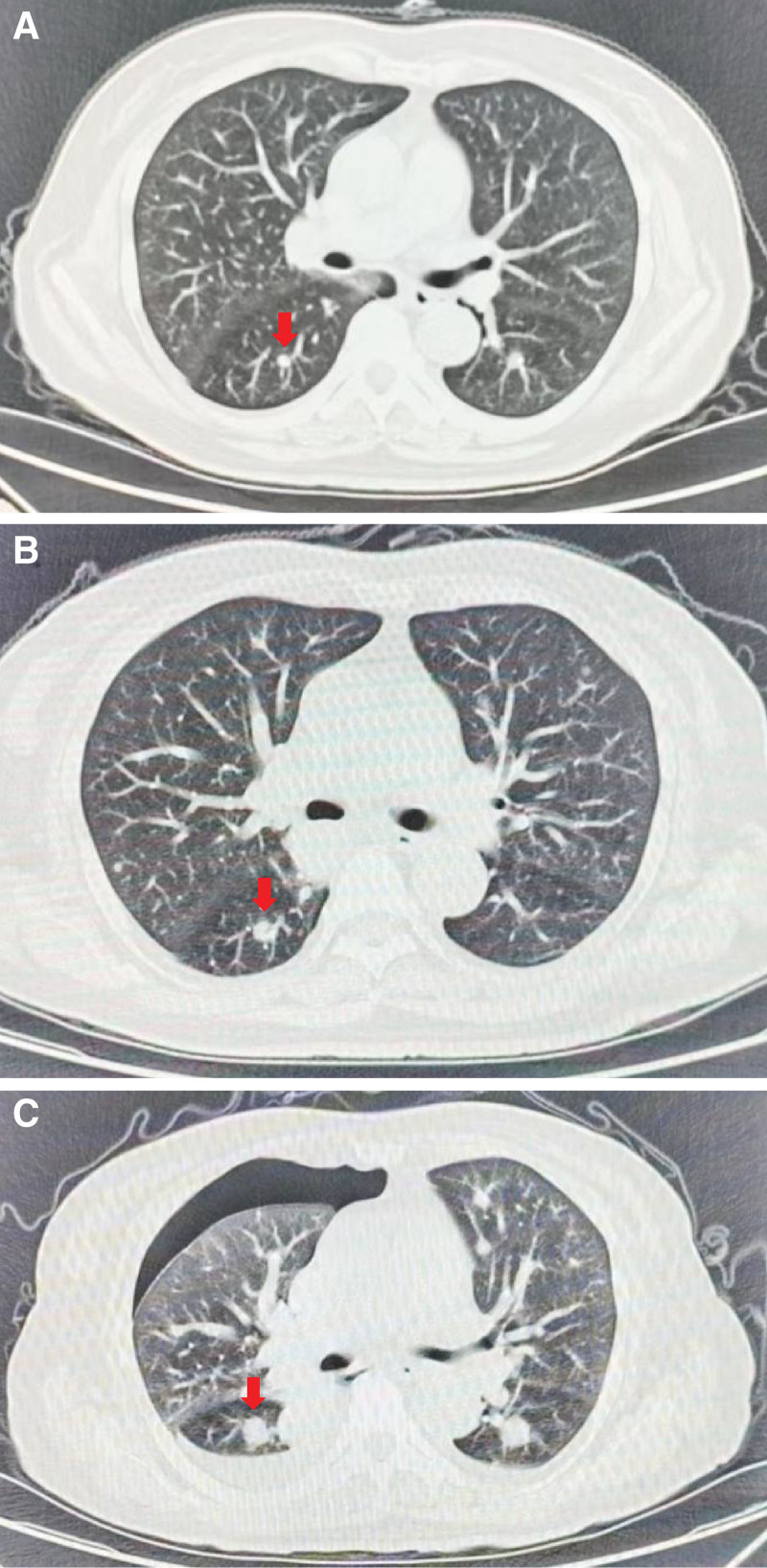
Computed tomography (A–C) shows multiple solid-density nodular shadows in both lungs, with the larger ones located in the dorsal segment of the right lower lobe and progressively enlarged (red arrow). (A) (2 days before surgery): the size of the pulmonary nodule is approximately 16 × 14 mm (Se203im28), and the boundary remains clear. (B) (11 days after surgery): the size of the pulmonary nodule is approximately 25 × 23 mm (Se203im23), and the boundary remains clear. (C) (24 days after surgery): the size of the pulmonary nodule is approximately 34 × 24 mm (Se203im24), and the boundary remains clear.

A 61-year-old postmenopausal woman arrived at the hospital on November 15, 2023, after experiencing abdominal pain for over a month and persistent fever for over 2 weeks. Chest computed tomography (CT) conducted on the same day revealed multiple solid nodules and small pleural effusions in bilateral lungs. Laboratory examination revealed slightly increased CA125 levels (38.9 U/mL; reference range: 0–35 U/mL). After admission to the gynecology ward, additional laboratory examinations indicated no abnormalities in human epididymis protein 4, CA199, carcinoembryonic antigen, or alpha-fetoprotein levels. A follow-up abdominal CT, performed 3 days before surgery, showed an irregular slightly low-density mass that measured 9.8 × 7.9 × 7.2 cm on the left side of the uterus of the patient (Fig. 1). Subsequent chest CT revealed new and slightly enlarged nodules compared with the previous ones (Fig. 2), indicating the high possibility of ovarian/fallopian tube neoplasms or peritoneal neoplasms with metastasis.

**Figure 3.**
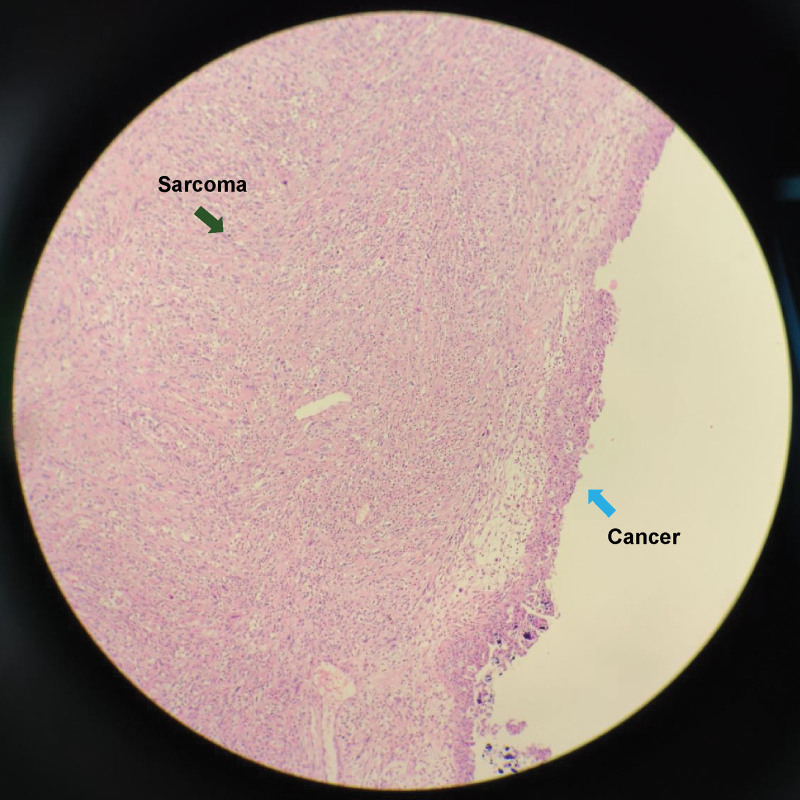
Ovarian carcinosarcoma. The left side contains sarcoma components (green arrow), and the right side contains cancer components (blue arrow) (hematoxylin and eosin staining × 100).

**Figure 4.**
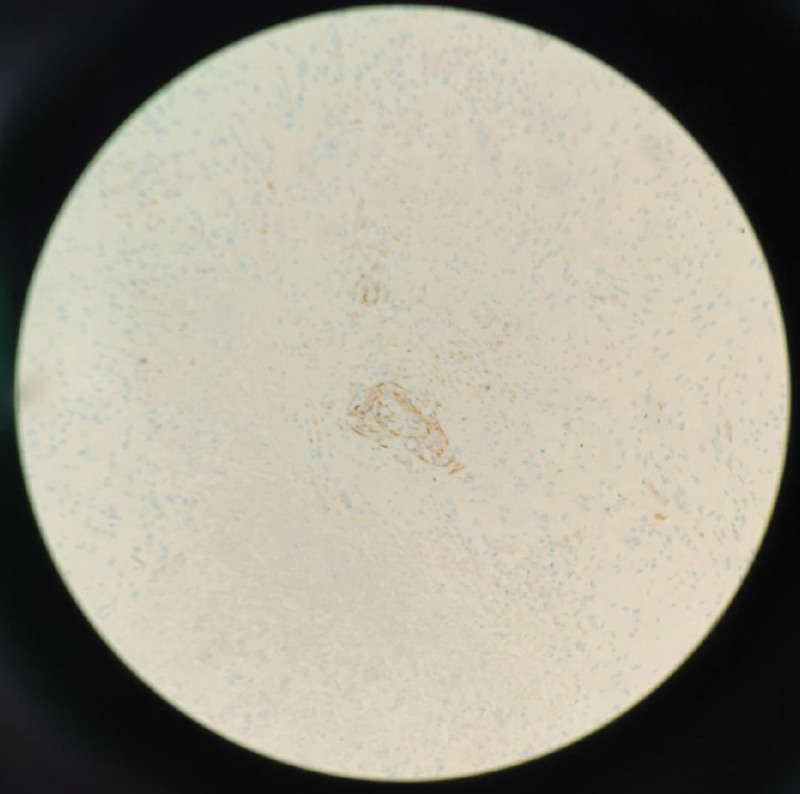
Immunohistochemistry×200; broad-spectrum cytokeratin staining showing positive adenocarcinoma components. Among them, the central brown area indicates cancer, and the surrounding area indicates sarcoma.

The patient underwent cytoreductive surgery based on laparoscopy results, and all detectable cancerous tissue was eliminated. Thoracoscopic surgery or biopsy of the small chest nodules was performed, as necessary. Pathological examination revealed a left OCS 10.0 cm in size with extensive necrosis (Figs. 3 and 4). The patient was postoperatively diagnosed with stage IVB OCS, based on the International Federation of Gynecology and Obstetrics guidelines.

The patient’s body temperature increased intermittently (≤37.5°C) after postoperative day 2 and increased significantly (36°C–39.5°C) after postoperative day 11, displaying an irregular fever pattern. Despite the elevated inflammatory markers, blood, sputum, and ascites cultures were negative, and serology for endotoxins, mycoplasma, influenza, mycobacteria, cytomegalovirus, and *Aspergillus* was unavailable. Postoperative chest CT revealed the appearance of new nodules that were slightly enlarged, along with a renewed increase in the pleural effusion (Fig. 2). Despite receiving empiric antibiotic therapy for at least 7 days, there was no obvious change in body temperature.

The case was reviewed in a multidisciplinary team meeting, and a pulmonary abscess was considered for the first time because of persistent fever and the rapid growth rate of pulmonary nodules. Additional laboratory examinations were conducted, and potent broad-spectrum anti-infection treatments were administered. However, C-reactive protein (CRP), rheumatoid factor, antinuclear antibody, and anti-streptolysin O test results were negative, and the patient’s high fever did not resolve. Subsequently, indomethacin was administered to effectively control the body temperature during the treatment period. A biopsy of the lung nodules was performed; however, this was terminated because puncture of the useful tissue was not achieved after postoperative day 19. Eventually, lung metastasis was considered owing to the patient’s history of OCS. Due to OCS with multiple lung lesions and increased creatinine levels (200 µmol/L), the patient received adjuvant chemotherapy with paclitaxel (300 mg) and gemcitabine (1.6 g) on postoperative day 21. The patient experienced progressive dyspnea on postoperative day 24, and an urgent chest CT showed emerging right pneumothorax (Fig. 2). After closed thoracic drainage, the symptoms gradually improved. The level of brain natriuretic peptide gradually increased (1238 pg/mL), and electrocardiography showed frequent atrial premature beats and aberrant ventricular conduction after postoperative day 49. Owing to the significant worsening of the patient’s condition, she was admitted to the intensive care unit for further treatment on January 14, 2024. The patient’s creatinine level was elevated compared with that before the procedure, with a reduction in urination and brain natriuretic peptide level > 30000.00 pg/mL. The patient subsequently died on January 18, 2023 (Table 1).

**Table 1:** Timeline of events.

Event	Date
Seeking medical attention because of symptoms	November 15, 2023
Cytoreductive surgery	November 24, 2023
Considerable increase again in body temperature	December 05, 2023
Treatment with indomethacin	December 07, 2023
Biopsy of the lung nodules	December 13, 2023
Adjuvant chemotherapy with paclitaxel and gemcitabine	December 15, 2023
Pulmonary nodules are larger than before	December 18, 2023
Frequent atrial premature beats and aberrant ventricular conduction	January 12, 2024
Declaration of death	January 18, 2024

This study was approved by the Medical Ethics Committee of Affiliated Hospital of Shandong Second Medical University (No. wyfy-2024-qt-025). The patient’s husband provided written informed consent for the publication of this case report and accompanying images.

## 3. Discussion

The definition of FUO calls for a fever lasting >3 weeks, multiple body temperature measurements >38.3°C, and the inability to obtain a diagnosis following a series of required investigations after 1 week of hospitalization.^[6]^ This is a diagnostic challenge for clinicians who are often unable to identify the etiology through systematic examination; thus, treatment relies on empirical medication. Although recent evidence supports that over half of the patients with FUO can be diagnosed using 18F-fluorodeoxyglucose positron emission tomography, which has a high sensitivity and relative specificity,^[7]^ its clinical application remains limited. Cancer accounts for approximately 2%–25% of FUO cases. According to Toussaint et al,^[8]^ 67% of cases of fever in patients with cancer are caused by infection, whereas 27% of noninfectious fevers are caused by the tumor itself.

NF, a paraneoplastic syndrome, is a frequent cause of FUO in patients with cancer. The pathophysiological mechanism of NF is related to the release of pyrogenic cytokines, particularly interleukin-1, interleukin -6, tumor necrosis factor (TNF)-α, interferon, and other miscellaneous factors.^[9]^ These cytokines stimulate the preoptic hypothalamus and induce the release of prostaglandin E2.^[10]^ Subsequently, fever is caused by the disinhibition of presympathetic neurons in the brainstem owing to prostaglandin E2 binding in the preoptic hypothalamus.^[11]^ The release of TNF-α from tumor necrotic tissue is also a pathophysiological mechanism of NF. Zhou et al^[12]^ reported that NF occurs in patients with lymphoma, leukemia, multiple myeloma, and renal cell carcinoma. If all other possibilities are excluded, NF can be considered. Owing to the positive effect of antibiotic administration on the prognosis of infected patients, timely differentiation between infectious fever and NF in patients with cancer is crucial. Infectious fever typically presents with chills and periodic sweating. Although sweating is a typical symptom of NF, chills are rare. Follow-up CRP levels significantly decrease in patients with cancer and infectious fever.^[13]^ Furthermore, the procalcitonin/CRP ratio is deemed to be the best marker for discriminating between infectious fever and NF.^[14]^

We report a unique case of lung metastatic OCS presenting as NF; the case conformed to the proposed diagnostic criteria for tumor fever.^[15]^ The various situations described above are consistent with the characteristics of NF. Tumor-induced fever may be palliated after surgery and adjuvant therapy; nonsteroidal anti-inflammatory drugs are also effective. Chang and Gross^[16]^ first reported the naproxen test as a traditional method for differentiating between NF and infectious fever. When naproxen was used to treat NF, the success rate was 94.1%, and when the dosage was increased to 250 mg twice daily, the success rate was 98.1%.^[17]^ NF can also be effectively treated with ibuprofen, rofecoxib, diclofenac, indomethacin, and other nonsteroidal anti-inflammatory drugs.^[18]^ Nevertheless, NF recurs in some patients if nonsteroidal anti-inflammatory drugs are discontinued after short-term treatment. Therefore, the treatment of NF is based on prompt detection and active treatment of the primary disease.

Accessory examinations can help differentiate OCS; however, they do not provide a definitive diagnosis. For instance, measuring elevated levels of CA125 is not of significant importance for evaluating the prognosis.^[19]^ The majority of OCS cases are characterized as masses > 10 cm, mostly made of solid materials, and are frequently accompanied by cystic degeneration and extensive hemorrhage and necrosis. The ultrasound finding that raises the most suspicion is that of large solid tumors with uneven margins and inhomogeneous echogenicity of the solid tissue and cystic areas.^[20]^ The accessory examinations described above can help differentiate OCS; however, they are not the source of a definitive diagnosis. The possibility of OCS should be considered in the differential diagnosis, especially when there are signs of empty blood vessels on magnetic resonance imaging.^[21]^

However, owing to the rarity of OCS in clinical practice, there are currently no standardized treatment regimens. Cytoreductive surgery remains the primary treatment strategy, with platinum-based chemotherapy being the second best.^[22]^ Compared with individuals treated with ifosfamide/paclitaxel, those treated with carboplatin/paclitaxel had a longer median progression-free survival.^[23]^ Subsequently, after more than 20 years of observation, a large retrospective case–control study reported by Heinzelmann-Schwarz et al^[24]^ unequivocally demonstrated that when anthracyclines and carboplatin were combined, OCS responded more favorably than when taxanes were used in combination with carboplatin (73.9% vs. 39.4%, respectively). Currently, strategies involving programmed cell death protein 1, programmed cell death 1 ligand 1, vascular endothelial growth factor, poly ADP-ribose polymerase, cytotoxic T-lymphocyte antigen-4, mechanistic target of rapamycin, or complement 3 inhibitors have shown promise when used alone or in combination; however, their effectiveness remains unclear.^[22]^

In summary, during diagnosis, OCS may present as FUO. If complete cytoreduction and other treatment measures have been taken but the fever does not respond and progressive enlargement of other organ lesions is found, NF caused by distant tumor metastasis can be considered after excluding infectious factors. Nonsteroidal anti-inflammatory drugs have differential and therapeutic significance. During checkups for FUO, clinicians should consider this rare diagnosis and pay attention to identifying the cause of the fever in a timely manner. Simultaneously, we encourage clinicians to continue exploring the optimal chemotherapy strategy for OCS as well as evaluating various nonsteroidal anti-inflammatory drugs to differentiate the cause of fever and establish the optimal dosage for fever treatment.

## Acknowledgments

We would like to thank Editage (www.editage.cn) for English language editing.

## Author contributions

**Conceptualization:** Weijing Wang, Xuechao Ji.

**Data curation:** Weijing Wang, Xinbo Wang.

**Writing – original draft:** Weijing Wang, Xuechao Ji.

**Resources:** Hanchao Yang.

**Writing – review & editing:** Hanchao Yang, Xinbo Wang.

**Funding acquisition:** Xinbo Wang.
